# Anxiety, depression, and stress in caregivers of children and adolescents with mental disorders in Ghana and implications for medication adherence

**DOI:** 10.4314/gmj.v55i3.1

**Published:** 2021-09

**Authors:** Patience ME Ocansey, Irene A Kretchy, Genevieve C Aryeetey, Kofi Agyabeng, Justice Nonvignon

**Affiliations:** 1 Department of Social and Behavioural Sciences, School of Public Health, University of Ghana, Legon, Ghana; 2 Department of Pharmacy Practice and Clinical Pharmacy, School of Pharmacy, University of Ghana, Legon, Ghana; 3 Department of Health Policy, Planning and Management, School of Public Health, University of Ghana, Legon, Ghana; 4 Department of Mathematics, KU Leuven, Leuven-Belgium

**Keywords:** Anxiety, depression, medication adherence, psychological burden, stress

## Abstract

**Objective:**

This study assessed levels of anxiety, depression, and stress among family caregivers of children and adolescents with mental disorders in Ghana and the implication on medication adherence.

**Design:**

A cross-sectional study.

**Setting:**

The study was conducted at the outpatient departments of the three main public psychiatric hospitals in Ghana.

**Participants:**

Two hundred and ten non-paid family caregivers of children and adolescents with mental disorders were recruited for this study.

**Main Outcome Measure:**

The study assessed symptoms of anxiety, depression and stress among the caregivers and estimated caregiver-reported medication adherence.

**Results:**

About 56.2%, 66.2% and 78% of the caregivers experienced severe anxiety, severe depression and moderate to severe stress symptoms respectively. From the multiple logistic regression model, while anxiety was significantly affected by religion and education, depression was influenced by sex, age, marital status, proximity to facility, and employment status. Female caregivers had about four times higher odds of being depressed compared to male caregivers (aOR: 3.81, 95% CI: 1.66 – 8.75). The caregiver-reported medication adherence was 11.9%. Anxiety was significantly predictive of medication adherence.

**Conclusion:**

Most family caregivers of children and adolescents with mental disorders experienced symptoms of anxiety, depression and stress with anxiety having implications for medication adherence. The study findings underscore the need to consider psychological characteristics of caregivers and the provision of mental health support for them, as part of the routine health care for children and adolescents with mental disorders.

**Funding:**

None indicated

## Introduction

Child and adolescent mental disorders (CAMD) are common, and a major cause of disease burden.^1^ The World Health Organization estimates 10 – 20% of children and adolescents experience mental disorders which are usually characterized by abnormalities in thoughts, emotions, behaviour and relationships with others.^2^, ^3^ To avoid mental disorders extending to adulthood and causing significant impairment in physical and mental wellbeing, addressing mental health needs is crucial. However, mental health services for CAMD, especially in low- and middle-income countries have been associated with challenges such as lack of availability and accessibility to child and adolescent mental health (CAMH) facilities^4^, inadequate CAMH professionals 5 and poor implementation of CAMH policies.^6^

Psychosocial interventions and pharmacotherapy are employed in managing CAMD, with evidence to show that pharmacological treatments are effective in many neuropsychiatric disorders.^7^ However, non-adherence to these medications have been reported and this results in negative mental and physical health outcomes.^7^,^8^

Adhering to medications is important for CAMH outcomes. However, only about 66% (±20.9%) of persons with CAMD adequately take their medications. Sub-optimal adherence has negative effects and several factors have been indicated for this behavior including caregiver anxiety and stress.^7^, ^8^, ^9^

While it is important to receive adequate formal care through services provided by healthcare institutions, CAMD are dependent on family caregivers. Family caregivers are persons with substantial emotional attachment to patients and are usually non-paid family members who offer care, emotional and physical support for their dependents.^10^ The caregivers play vital roles in the child's development, and supervise pharmacological and nonpharmacological interventions that may be introduced by health providers within the formal healthcare setting. 9

The caregiving process is often a time consuming and emotionally draining experience associated with stresses which negatively impacts on the caregiver, family routines and the patients. Children with mental disorders require supervision in their daily activities, and this process can affect the physical and mental health of the caregiver.^11^ Yet, these caregivers tend to disregard their personal health needs leading to increased caregiver burden as a result of the psychological distress they experience. 12

Burden of care in mental health has been associated with difficulties and/or adverse events that the caregiver experiences by exhibiting symptoms of anxiety, depression and stress which may be manifested in feelings of loneliness, distress and the tendency to be upset easily due to the demands of caregiving.^9^, ^13^ The increased burden and poor psychological wellbeing tend to negatively affect caregiver lifestyle, relationships and roles including medication-related and adherence responsibilities where caregivers administer or supervise medicines. 9 An emotionally distressed caregiver may forget to administer or poorly supervise routine medication use which may lead to negative health outcome.^6^

Existing literature have mainly focused on caregiving and adherence in adult psychiatric populations and there remains a dearth of information on caregiver psychological characteristics and associated risk to adherence in paediatric populations with mental disorders. This study, therefore, sought to estimate levels and determinants of anxiety, depression and stress of caregivers of CAMD, and assess the impact on medication adherence.

## Methods

### Study design and setting

The study employed a cross-sectional design in the three main psychiatric hospitals in the country- Accra and Pantang Hospitals in the Greater Accra Region of Ghana and Ankaful Psychiatric Hospital in the Central Region. The Ankaful Psychiatric Hospital was established in 1965. It is in Ankaful, a suburb of the Central Region. The Hospital has about eleven wards and serves the Central and other neighboring regions in western and northern Ghana. Pantang and Accra Psychiatric Hospitals began operations in 1960 and 1905 respectively. The former has about 500 bed capacity while the latter has around 600 bed capacity. Both facilities provide services to patients in Ghana and Togo, Burkina Faso, and Ivory Coast. Conditions reported and managed in all three facilities include neurodevelopmental disorders such as autism, intellectual disabilities, elimination disorders, attentiondeficit and disruptive behaviour disorders, as well as, anxiety, mood, substance use, schizophrenia spectrum and other psychotic disorders.^14^–^17^

### Study population and sampling

The study population comprised family caregivers of CAMD who reported to the outpatient departments of all three facilities. Caregivers were eligible for the study if they were 18 years or above, unpaid and live with the child for a minimum of one year. Participants were excluded if they did not consent for participation although meeting the eligibility criteria. The estimated sample size for the three facilities was calculated using the Cochrane formula for sample size determination.^18^ An estimated proportion of caregivers with psychological distress of 38.3% 19 was used with 5% margin of error and 10% nonresponse rate. Using the proportional sampling approach, the sample size was distributed across the three psychiatric facilities based on a total of 2,180 children and adolescents who were managed in Accra (1317), Pantang (118) and Ankaful (745) Psychiatric Hospitals respectively and based on the assumption that these patients were accompanied by their caregivers. A total of 126, 12 and 72 caregivers for the three facilities respectively accounted for the estimated sample of 210.

With assistance from the nurses at the OPD of the various facilities, awareness was created about the study at the time when routine announcements for each day to patients and caregivers visiting the hospital was being made. Based on the medical records, the nurses assisted in identifying potential patients whose caregivers were likely to meet the inclusion criteria for the study. The potential accompanying caregivers were then contacted for possible recruitment into the study while waiting for the patient's scheduled appointment. Caregivers who agreed to be enrolled then endorsed the informed consent forms. Participants were continuously recruited in same manner during the recruitment period until the desired sample size per facility was reached.

### Data collection

Caregivers were interviewed using a structured questionnaire. Psychological burden was determined using recognized tools for measuring anxiety, depression and stress and medication adherence was assessed based on the caregiver-reported estimates.

#### Anxiety

Anxiety was measured using the 21-item Beck Anxiety Inventory (BAI). The BAI is used in estimating the severity of anxiety in both children and adults.^20^ The scale is scored from 0 to 3 and total scores categorized as 0–7 for normal to minimal anxiety, 8–15 for mild to moderate anxiety, 16–25 for moderate to severe anxiety and 26–63 for severe anxiety. The Cronbach coefficient alpha of the BAI was 0.93 in a previous study 21 and 0.88 in this study.

#### Depression

The Beck Depression Inventory (BDI) measured depression among the caregivers. 22 This is a 21-item self-report inventory consisting of symptoms on cognitions of guilt, hopelessness and irritability, and physical symptoms like weight loss and fatigue. Each question was rated on a 4-point Likert scale with total rating categorized into minimal (0–13), mild (14–19), moderate (20–28) and severe (≥ 29). The BDI has been previously used to measure depressive symptoms in some developing countries and found to have adequate reliability.^23^ In this study, reliability coefficient was 0.95.

#### Stress

Parental Stress Scale (PSS) was used in measuring the stress levels of the caregivers. It is an 18-item self-report scale developed by Berry and Jones (1995) 24 as a substitute to the 101-item Parenting Stress Index measured on a 5 – Point scale (1-strongly disagree, 2-disagree, 3-undecided, 4-agree, and 5-strongly agree). This tool measures changes in stress levels of carers. The total scores ranged from 18 to 90 and higher scores signified higher levels of stress. The reliability of the PSS has been established in similar cultures 25 and in this study, a reliability coefficient of 0.71 was noted.

#### Medication adherence

The Medication Adherence Report Scale (MARS-5) was adapted to assess the caregiver-reported adherence to medicines. The questions were modified to solicit for caregivers' ability to consistently give their wards their medication as prescribed. For instance, the 1^st^ item on the scale that reads “I sometimes forget to take my medicine” was modified to “I sometimes forget to give my ward his/her medicine”. Total scores ranged from 5 to 25 and dichotomized into adherent if MARS score was 25 or non-adherent (< 25). Reliability coefficient of the scale in this study was 0.61 with a previous study establishing the reliability of the use of MARS among caregivers in Ghana.^9^

Data from the caregivers were collected using a researcher-administered, pencil-and-paper based quantitative survey which lasted approximately 20 minutes. The interviews were conducted primarily in English, and two other Ghanaian languages Ga and Twi/Fante, by trained research assistants who were proficient in these languages. The questionnaire was modified to incorporate translations of terms from English to the local languages and these were back translated into English by language experts. Before the final administration of the questionnaire, it was pre-tested among 20 participants and the tool was generally found to be reliable.

### Data analysis

Data entry was done in SPSS version 22 and exported into STATA version 15 for analysis. Descriptive statistics of categorical variables were reported as frequencies and percentages while means and standard deviations were reported for continuous variables. Test of normality was done with a histogram, standardized normal probability plot and Skewness/Kurtosis tests for normality. The levels of depression and anxiety were further dichotomized into (“0” No – minimal and mild, “1” Yes – Moderate and Severe). Chi-square tests of independence was used for associations between categorical background characteristics and binary outcome variables. Welch ttest and one-way ANOVA test were used in comparing average stress scores between categories and more than two categories respectively. Multiple binary logistic regression models were used to assess the effects of the caregiver characteristics on depression, anxiety, and adherence outcome variables while multiple linear regression model was used for stress. Adjusted odds ratio and linear regression coefficient with 95% confidence interval and p-values were reported. All statistical tests of significance were measured at 5%.

### Ethical Approval

Ethical approval for this study was granted by the Ghana Health Service Ethical Review Committee (GHS-ERC065/02/19). Each respondent signed a consent form prior to data collection. Each participant was anonymised to protect their identity. Health practitioners who served as contact persons were notified about distressed participants for follow up supportive care.

## Results

### Socio-demographic characteristics of caregivers

Caregivers involved in the study were 210. The majority (66.7%, 140/210) were females. Averagely they were aged 33.9 ± 10.3 years old with 72.4% (152/210) never married/ single. Christianity was the dominant religious affiliation and the majority (52.95, 111/210) had tertiary level education. Most of the caregivers were employed (Table 1).

**Table 1:** Socio-demographic characteristics of caregivers

Variables	Number (%)
**Sex**	
**Male**	70 (33.3)
**Female**	140 (66.7)
**Age (years)**	
**Mean ± SD**	33.9 ± 10.3^#^
**20–29**	71 (33.8)
**30–39**	82 (39.0)
**40–49**	39 (18.6)
**≥50**	18 (8.6)
**Marital status**	
**Never Married**	152 (72.4)
**Currently Married**	42 (20.0)
**Formerly Married**	16 (7.6)
**Place of residence (Perceived distance)**	
**Closer to the facility**	96 (45.7)
**Far from the facility**	114 (54.3)
**Religion**	
**Christian**	160 (76.2)
**Non-Christian**	50 (23.8)
**Highest level of education**	
**No education**	25 (11.9)
**Primary education**	22 (10.5)
**Junior High School**	14 (6.7)
**Secondary/ High School**	38 (18.1)
**Tertiary cert/diploma/post diploma**	111(52.9)
**Employment status**	
**Unemployed**	28 (13.3)
**Self-employed**	46 (21.9)
**Private sector**	12 (5.7)
**Public sector**	81 (38.6)
**Student/apprentice**	43 (20.5)

### Levels of Anxiety, depression, stress and reported medication adherence

Average anxiety score was 27.0 ± 10.8 (Range: 0 – 57) with 56.2% (118/210) experiencing severe anxiety. Average depression score was 32.2 ± 14.9 (Range: 0 – 57) and about two-thirds (66.2%, 139/210) experienced severe depression. The average stress score was 50.67 ± 7.6 (Range: 34 – 72) with 78% (163/210) experiencing moderate to severe stress. Average medication adherence score was 20.9 ± 3.3 (Range: 9 – 25) with optimal adherence of 11.9% (25/210) reported (Table 2).

**Table 2:** Levels of anxiety, depression, stress and reported medication adherence

Variables	Number (%)
**Anxiety**	
**Mean ± SD**	27.0 ± 10.8^#^
**Minimal**	8 (3.8)
**Mild**	20 (9.5)
**Moderate**	64 (30.5)
**Severe**	118 (56.2)
**Depression**	
**Mean ± SD**	32.2 ± 14.9^#^
**Minimal**	34 (16.2)
**Mild**	16 (7.6)
**Moderate**	21 (10.0)
**Severe**	139 (66.2)
**Stress**	
**Mean ± SD**	50.67 ± 7.6^#^
**Low to moderate**	47 (22.4)
**Moderate to severe**	163 (77.6)
**Medication Adherence**	
**Mean ± SD**	20.9 ± 3.3^#^
**Non-Adherence**	185 (88.1)
**Adherence**	25 (11.9)

Age, place of residence, education, and employment were significantly associated with depression (p<0.05). Depression was more prevalent among females than males (82.1% vs 64.3%). The prevalence of depression was lower among those with some level of formal education than those with no formal education. Caregivers staying at a perceived far distance from the health facility recorded lower prevalence of depression compared to those living closer to the facility (69.3% vs 69%, p=0.011). From the ANOVA tests, the average stress was found to be associated with marital status (p=0.047) and employment status (p=0.008). Medication adherence was significantly associated with sex, age, marital status, education, anxiety, and depressive symptoms (p<0.05). Higher levels of depression and anxiety were associated with lower proportion of adherence (Table 3).

**Table 3:** Association between background characteristics of caregivers and levels of anxiety, depression, stress and reported medication adherence

	Anxiety		Depression		Stress		Medication Adherence	
	Yes, n (%)	p-value^§^	Yes, n (%)	p-value^§^	Mean ±SD	p-value^¥^	Yes, n (%)	p-value^§^
***Sex***		0.774		**0.004**		0.788		**0.01**
*Male*	60 (85.7)		45 (64.3)		50.5 ± 7.2		14 (20.0)	
*Female*	122 (87.1)		115 (82.1)		50.8 ± 7.8		11 (7.9)	
***Age (years)***		**0.01**		**0.016**		0.6827		**0.041**
*20–29*	65(91.6)		58(81.7)		49.8 ± 7		7(9.9)	
*30–39*	67(87)		55(71.4)		50.7 ± 8.3		8(10.4)	
*40–49*	34(87.2)		33(84.6)		51.3 ± 7.4		4(10.3)	
*≥50*	11(61.1)		9(50)		51.8 ± 8.3		6(33.3)	
***Marital status***		0.054		0.076		**0.047**		**0.006**
*Never Married*	137(90.1)		122(80.3)		49.9 ± 7.7		12(7.9)	
*Currently Married*	33(78.6)		28(66.7)		51.9 ± 6.8		8(19.1)	
*Formerly Married*	12(75)		10(62.5)		54.3 ± 7.8		5(31.3)	
***Place of residence (Perceived*** ***distance)***		0.050		**0.011**		0.599		0.299
*Closer to the facility*	88(91.7)		81(84.4)		50.4 ± 7.5		9(9.4)	
*Far from the facility*	94(82.5)		79(69.3)		50.9 ± 7.8		16(14)	
***Religion***		0.081		0.137		0.4493		0.634
*Christian*	135(84.4)		118(73.8)		50.4 ± 7.8		20(12.5)	
*Non-christian*	47(94)		42(84)		51.4 ± 7.2		5(10)	
***Highest level of education***		**<0.001**		**0.041**		0.085		**0.006**
*No education*	25(100)		24(96)		51.2 ± 7.1		1(4)	
*Primary education*	15(68.2)		17(77.3)		52.7 ± 8.4		4(18.2)	
*Junior High School*	10(71.4)		11(78.6)		49.8 ± 7.9		1(7.1)	
*Secondary/ High School*	29(76.3)		24(63.2)		53 ± 7.2		11(29)	
***Tertiary cert/diploma/post*** ***diploma***	103(92.8)		84(75.7)		49.5 ± 7.5		8(7.2)	
***Employment status***		0.086		**0.045**		**0.008**		0.573
*Unemployed*	44(95.7)		39(84.8)		52 ± 7.9		6(13)	
*Self-employed*	8(66.7)		7(58.3)		54.4 ± 7.1		3(25)	
*Private sector*	70(86.4)		61(75.3)		49.7 ± 7.6		10(12.4)	
*Public sector*	24(85.7)		25(89.3)		53.5 ± 6.2		2(7.1)	
*Student/apprentice*	36(83.7)		28(65.1)		48.3 ± 7.4		4(9.3)	
***Anxiety***								**<0.001**
*Minimal*	NA		NA		NA		4(50)	
*Mild*	NA		NA		NA		4(20)	
*Moderate*	NA		NA		NA		13(20.3)	
*Severe*	NA		NA		NA		4(3.4)	
***Depression***								**0.001**
*Minimal*	NA		NA		NA		8(23.5)	
*Mild*	NA		NA		NA		5(31.3)	
*Moderate*	NA		NA		NA		4(19.1)	
*Severe*	NA		NA		NA		8(5.8)	
***Stress (Mean ± SD)***	NA		NA		NA		50.4±7.8 vs 52.3±6.1	0.180

### Effect of caregiver characteristics on aanxiety, depression, stress and reported medication adherence

Results from the multiple logistic regression model revealed that females had about four times higher odds of being depressed compared with the male caregivers (aOR: 3.81, 95% CI: 1.66 – 8.75). Caregivers who were currently or formerly married had 76% or 63% lesser odds of being depressed than those who were never married. Non-Christians had more than five times higher odds of being anxious compared with those who were Christians (aOR: 5.1, 95% CI: 1.01 – 26.27). Caregivers with junior high school and secondary levels of education had higher odds of being anxious compared to those with primary education.

The odds of being depressed among caregivers with employment was at least 56% lower than those who were unemployed. From the adjusted linear regression model, none of the factors was significantly predictive of the mean stress level (p>0.05) (Table 4). Anxiety was the only factor that significantly predicted adherence. From the multiple binary logistic regression model, participants with mild, moderate, and severe levels of anxiety had 95%, 86% and 97% reduced odds of adherence compared to those with minimal anxiety (Table 4).

**Table 4:** Effect of background characteristics of caregivers on anxiety, depression, stress and reported medication adherence

	Anxiety		Depression		Stress		Medication Adherence	
	aOR (95% CI)	p-value	aOR (95% CI)	p-value	β (95% CI)	p-value	aOR (95% CI)	p-value
**Sex**		0.758		**0.002**		0.902		0.077
**Male**	1		1		0		1	
**Female**	1.18(0.4, 3.47)		3.81(1.66, 8.75)		0.14(-2.12, 2.4)		0.35(0.11, 1.12)	
**Age (years)**		0.238		**0.019**		0.994		0.297
**20–29**	1		1		0		1	
**30–39**	1.03(0.27, 3.9)		0.45(0.17, 1.22)		0.15(-2.55, 2.84)		0.46(0.11, 2.01)	
**40–49**	2.2(0.41, 11.83)		2.45(0.61, 9.78)		0.36(-2.95, 3.67)		0.16(0.02, 1.21)	
**≥50**	0.36(0.06, 2.03)		0.3(0.07, 1.38)		0.53(-3.83, 4.88)		0.61(0.06, 6.5)	
**Marital status**		0.144		**0.045**		0.366		0.315
**Never Married**	1		1		0		1	
**Currently Married**	0.25(0.06, 1)		0.24(0.08, 0.75)		0.7(-2.43, 3.83)		3.39(0.69, 16.54)	
**Formerly Married**	0.64(0.08, 4.81)		0.37(0.06, 2.19)		3.56(-1.39, 8.51)		2.5(0.27, 23.43)	
**Place of residence** **(Perceived distance)**		0.191		**0.001**		0.934		0.796
**Closer to the facility**	1		1		0		1	
**Far from the facility**	0.47(0.15, 1.45)		0.21(0.08, 0.51)		0.1(-2.19, 2.38)		1.19(0.31, 4.52)	
**Religion**		**0.049**		0.499		0.854		0.942
**Christian**	1		1		0		1	
**Non-Christian**	5.13(1, 26.27)		1.45(0.49, 4.25)		0.25(-2.45, 2.95)		1.06(0.25, 4.49)	
**Highest level of education**		**0.002**		0.252		0.828		0.218
**No education**	1 (empty)		1		0		1	
**Primary education**	1		0.27(0.02, 3.57)		0.97(-4.18, 6.13)		3.19(0.21, 48.45)	
**Junior High School**	1.14(0.16, 8.12)		0.54(0.03, 8.62)		-2.27(-8.24, 3.71)		0.73(0.02, 30.89)	
**Secondary/ High** ** School**	3(0.61, 14.78)		0.11(0.01, 1.39)		0.52(-4.34, 5.38)		4.62(0.29, 73.78)	
**Tertiary cert/diploma** **/post diploma**	18.43(3.75, 90.61)		0.31(0.03, 3.89)		0.05(-4.9, 5)		0.61(0.03, 11.23)	
**Employment status**		0.066		**0.006**		0.172		0.762
**Unemployed**	1		1		0		1	
**Self-employed**	2.24(0.32, 15.69)		0.44(0.08, 2.56)		-1.85(-5.95, 2.24)		1.77(0.23, 13.36)	
**Private sector**	0.2(0.03, 1.54)		0.15(0.02, 1.22)		0.6(-5.1, 6.29)		3.2(0.19, 53.48)	
**Public sector**	0.2(0.04, 1.14)		0.19(0.03, 1.12)		-3.95(-8.27, 0.36)		4.37(0.47, 40.45)	
**Student/ apprentice**	0.22(0.04, 1.13)		0.05(0.01, 0.29)		-4.59(-8.97, -0.21)		2.44(0.18, 33.05)	
**Anxiety**								**0.037**
**Minimal**	NA		NA		NA		1	
**Mild**	NA		NA		NA		0.05(0, 0.93)	
**Moderate**	NA		NA		NA		0.14(0.01, 2.52)	
**Severe**	NA		NA		NA		0.03(0, 0.66)	
**Depression**								0.908
**Minimal**	NA		NA		NA		1	
**Mild**	NA		NA		NA		1.16(0.14, 9.44)	
**Moderate**	NA		NA		NA		1.32(0.19, 9.46)	
**Severe**	NA		NA		NA		0.7(0.12, 3.99)	
**stress**	NA		NA		NA		1.03(0.95, 1.12)	0.458

aOR: Adjusted odds ratio from multiple binary logistic regression model, β : Adjusted linear regression model coefficient, CI: Confidence interval, empty: predicts success perfectly

## Discussion

Caregiving for persons with chronic conditions such as mental disorders is a daunting task that is likely to have physical and psychological impact. This study sought to determine the psychological burden of caregiving for CAMD and the implication on medication adherence in the three major mental health facilities in Ghana.

### Anxiety, depression, and stress in caregivers

Our study found that around 75% of caregivers experienced moderate to severe anxiety, similar to a review conducted by Sherer et al, (2019). 27 Using the Zarit Burden Interview, Panday and Shamar reported caregivers' experienced severe to moderate burden with anxiety being associated with the burden.^28^ Caregivers tend to worry about the declining conditions of their wards.

The high levels of anxiety could be attributed caregivers being worried about the wellbeing of their children or the constant reminder of having a child with mental disorders.^11^, ^19^

Having a ward with mental disorders frequently requires a reorientation and re-examination of family objectives, duties and connections and this can negatively impact on the emotions of the caregiver which could lead to depression or anxiety. 11 Caregivers may be discouraged and depressed.^29^–^31^

### Caregivers' characteristics on anxiety, depression and stress

Caregivers' social and demographic characteristics were associated with burden. 32 In our study, most caregivers were females. Female caregivers are a common phenomenon for reasons including being mothers, having compassion and society's perception of women being better at providing care to persons with chronic conditions. 33 Women have been reported to have high levels of stress, anxiety and depression compared to men when caring for CAMD, 34 and compared to this study, depression was key.

Age was significantly associated with depression, more specifically, those within the 40–49 years range were 2.45 times more likely to experience depression compared to those within the 20–29 years range while caring for CAMD. The evidence on the relationship between caregivers' age and depression is mixed. While some observe increase in depression among older caregivers 35 others found younger caregivers to be more depressed. 36

This study showed that being previously or currently married was associated with lesser odds of having depression, indicating that people living together may be able to share and encourage each other in times of emotional hardships. Thus, it could happen that depression and even the other emotional experiences are likely to be minimized with support from significant others.

Again, caregivers are depressed because they are not assured of when their wards may get better. These health concerns can also lead to poor sleeping patterns for the caregivers and this may also account for the symptoms of depression that caregivers experience. 29 This study thus corroborates with similar studies that have indicated that caregivers usually experienced moderate to severe forms of depression while caring for their wards. 23

Stress is a common psychological consequence of caregiving, particularly in caring for children and adolescents with mental disorders. This study showed that about 78% of caregivers experienced moderate to severe forms of stress. Plausible reasons for this are the overwhelming demands from having to pay continuous attention to the needs of the child in addition to washing, cooking, cleaning, and managing unexpected bouts from the children.^13^,^19^

Caregivers may seem to be in a perpetual cycle of working with limited time to rest or pay attention to their personal needs. Nonetheless, some caregivers may not perceive this overwhelming day to day activities of caring for their sick wards as burdensome, more as part of their responsibility to their wards. In this case, they may report mild to moderate levels of stress. Again, where caregivers receive additional support from other family members, the burden of stress is likely to be low. 30

Religious affiliations and obligations have been viewed to have influence on the emotional wellbeing of people. Many people particularly in developing countries have strong attachment towards their beliefs and obligations, which shape their physical and mental attitudes and approaches to life situations.^37^, ^38^ It has been observed that some religious practices may even change the functioning of the brain to improve mental health. 39 This study for example found that non-Christians were 5 times more likely to be anxious compared to Christians. Thus, people that are devout to a certain religious course may have fewer symptoms of anxiety and depression. For example, Fourohi *et al* in a systematic review, found that as religious orientation increased, anxiety and depression declined.^38^ On the other hand, some religious obligations (i.e., rituals and belief systems) may trigger or worsen mental conditions of patients and caregivers if not managed appropriately. 39

This study found that caregivers with higher education had higher odds of being anxious compared to those with primary education. This may seem so given the current ease of access to information. Caregivers can read about the content of any sickness online, some of which may not be censored. Where caregivers are able to assimilate much information regarding the mental conditions of their wards, this may cause them to be more anxious compared to being unaware of details of the conditions.

The study also showed that those who were self-employed were 2.2 times likely to experience anxiety compared to those who were unemployed. However, students, and those working in the public and private sectors had lessor odds of being anxious compared to the unemployed.

Plausible increase in odds of anxiety among the self-employed is that in a typical lower middle-income country such as Ghana, being self-employed means engaging with and competing for customers daily either for trading activities or provision of services. Thus, the regular routine of such persons is likely to be disrupted as well as the thought of losing customers due to the time spent in caregiving for their wards with mental illness, may increase anxiety among such persons in comparison to those in the formal sector with relatively stable employment. Similarly, caregivers in the various employment categories had lesser odds of being depressed compared to the unemployed. Being unemployed inherently places a person to be anxious or depressed compared to persons engaged in some stable economic activity.^40^

### Depression, anxiety and stress of caregivers and implications for medication adherence

The evidence on the reported medication adherence levels among caregivers of children and adolescents with mental disorders globally and Ghana in particular is limited. The study revealed the caregiver reported medication adherence among children with mental illness was about 12%, like other studies. 41 Adherence to medication, particularly in child and adolescent mental healthcare is important for improved quality of life of outcomes while addressing the appropriate use and risk of potential abuse for these pharmacological agents. 42, 43 Various factors are likely to influence adherence to medication including the psychological state of caregivers.

The current study showed that anxiety was the only psychological condition that influenced medication adherence, i.e., having mild, moderate or severe anxiety in comparison to minimal anxiety was associated with lower odds of medication adherence. Others have however found depression and anxiety to have an influence on medication adherence. 42 Reasons given among others were that usually caregivers, in most cases mothers were under pressure to balance childcare needs and household chores which affected the administration of medication to their wards. 44

It is important to note that because the focus of the study was on caregivers, the study acknowledges the limitation of not obtaining detailed clinical information on the children and adolescents from the health records which may be potential determinants of the study outcomes. Again, other social and economic characteristics of caregivers including relationship with patient, stigma, socio economic status, and ability to pay for medications out of pocket may be potential confounders to the study results. Causal associations of the variables can also not be ascertained given the limitations with cross-sectional study designs.

Our study is, however, strengthened in the fact that data from the caregivers were obtained from all the three major psychiatric health facilities in Ghana, thus making the findings relevant for clinical settings and highlighting the need to consider instituting some form of mental health support for caregivers as part of the routine care for children and adolescents with mental illness.

Prospects for further studies include exploring caregiver burden in other non-clinical or social settings.

## Conclusion

Most family caregivers of children and adolescents with mental disorders experienced symptoms of anxiety, depression and stress with anxiety having implications for medication adherence. The study findings underscore the need to consider psychological characteristics of caregivers and the provision of mental health support for them, as part of the routine health care for children and adolescents with mental disorders.
